# Growth and Metal Partitioning of the High-Biomass Grass Cultivar Minmu 6 Under Graded Cadmium and Lead Exposure

**DOI:** 10.3390/plants15193068

**Published:** 2026-10-08

**Authors:** Liuting Zhou, Xiaoyun Huang, Yafen Wu, Chengran Yu, Haidong Han, Deqing Feng, Dasong Lin, Xiusheng Huang

**Affiliations:** 1 Institute of Resources, Environment and Soil Fertilizer, Fujian Academy of Agricultural Sciences, Fuzhou 350013, China; zlt0616@126.com (L.Z.);; 2 Fujian Province Engineering and Technology Research Center for Recycling Agriculture in Hilly, Fuzhou 350013, China; 3 Agro-Environmental Protection Institute, Ministry of Agriculture and Rural Affairs, Tianjin 300191, China

**Keywords:** cadmium, lead, Minmu 6, phytoremediation, metal partitioning, biomass

## Abstract

Phytoremediation requires plants that combine high biomass production with tolerance to metal stress. The responses of *Pennisetum americanum* (L.) Leeke *× Pennisetum purpureum* Schumach. ‘Minmu 6’ were evaluated in separate pot experiments with Cd at 0 (CK), 1.5 (L-Cd), 10 (M-Cd), and 25 mg kg^−1^ (H-Cd), and Pb at 0, 600 (L-Pb), 1200 (M-Pb), and 1800 mg kg^−1^ (H-Pb). Growth, physiological traits, and organ-level metal concentrations were measured at 45, 90, and 135 d. Mantel tests explored associations among exposure conditions, tissue metal concentrations, and plant traits. Under the selected exposure regimes, Cd-treated plants showed larger reductions in shoot growth than Pb-treated plants. Both metals were concentrated in roots, with lower shoot-to-root concentration ratios for Pb than for Cd. At 135 d, whole-plant metal removal efficiencies at the highest Cd and Pb addition rates were 1.29% and 0.21%, respectively. These values include root-associated metal and therefore exceed the fractions potentially removable through shoot harvesting alone. The observed partitioning patterns identify shoot Cd removal and root-associated Pb retention as priorities for further evaluation; they do not establish distinct remediation functions. Practical performance remains to be tested in aged contaminated soils and field experiments, with direct measurements of harvestable metal removal and soil metal mobility.

## 1. Introduction

Soil contamination by heavy metals threatens safe land use, ecosystem restoration, and agricultural product quality. Cadmium (Cd) and lead (Pb) persist in soils and can inhibit plant growth, impair soil functions, and enter the food chain [1]. Shi et al. [2] reported substantial spatial and land-use-related variation in soil metal contamination across China. Contamination was generally greater in southeastern than in northwestern China, with notable Cd risks in agricultural soils and Pb-related health risks. These findings highlight the need to identify suitable remediation plants for acidic soils and mining-affected agroecosystems in southern China. Phytoremediation, the use of plants to remove or immobilize contaminants, offers an in situ approach with relatively low cost and limited soil disturbance. Phytoextraction removes metals through the harvesting of contaminated plant tissues, usually shoots, whereas phytostabilization limits metal mobility through root retention and immobilization in the rhizosphere, the soil zone influenced by roots [3]. However, many conventional hyperaccumulators have low biomass, slow growth, or limited environmental adaptability [4,5]. Plants that combine high biomass with metal tolerance and accumulation or immobilization capacity may therefore offer complementary remediation options.

High-biomass grasses have several traits that may support phytoremediation. *Pennisetum* species and hybrids often grow rapidly, develop extensive root systems, and regrow after cutting. Biomass production may compensate for moderate tissue metal concentrations, although removal must be evaluated from the metal content of harvested shoots [6]. Studies of hybrid elephant grass and hybrid *Pennisetum* have reported growth maintenance and Cd extraction under Cd-contaminated conditions. Their root systems may also retain metals belowground and limit transfer to shoots [7,8]. Minmu 6 is a cultivar derived from *Pennisetum americanum* (L.) Leeke *× Pennisetum purpureum* Schumach. and was developed by the Fujian Academy of Agricultural Sciences. These growth traits make it a candidate for investigating the balance between biomass maintenance, metal uptake, and organ-level partitioning. Reported fresh and dry biomass yields reach 230 and 27 t·hm^−2^, respectively [9]. These traits make Minmu 6 a candidate for evaluating metal accumulation, vegetation cover, and metal retention in the acidic red soils of southern China.

Although *Pennisetum* species and hybrids have been studied in metal-contaminated soils, most studies have focused on Cd alone or on total metal accumulation. Direct comparisons of Cd and Pb responses within the same high-biomass cultivar remain limited, particularly for physiological injury, organ-level metal partitioning, and potential remediation function. Cd is generally more phytoavailable, meaning more readily available for plant uptake, and more mobile within plants than Pb. It can disrupt photosynthesis, mineral nutrition, and antioxidant metabolism [6,10]. Pb is usually less readily translocated and is retained more strongly at root surfaces, in root cell walls, and in the rhizosphere [11,12]. These differences make metal-specific assessment essential. Total accumulation or a single enrichment or translocation index cannot fully describe the balance between uptake, retention, and harvestable removal. Oxidative stress, membrane damage, pigment changes, and osmotic adjustment may further influence tolerance and metal distribution [13,14,15]. Comparisons across growth stages are therefore needed to connect these physiological responses with biomass maintenance and organ-level metal partitioning.

To address these gaps, the present study compared the responses of Minmu 6 to graded Cd and Pb exposure at 45, 90, and 135 d. The working hypothesis was that Cd and Pb would show contrasting organ-level partitioning, with Cd exhibiting greater relative transfer to aboveground tissues and Pb showing stronger root association under the selected exposure regimes. Growth, biomass allocation, physiological traits, organ-specific metal concentrations, bioconcentration factors, translocation factors, and whole-plant metal removal efficiencies were evaluated to test these predictions. Exploratory Mantel analyses were used to examine covariation among exposure conditions, tissue metal concentrations, sampling time, and plant traits. The study was designed to characterize metal partitioning and to identify remediation-related traits requiring further validation rather than to assume distinct remediation functions a priori.

## 2. Results

### 2.1. Growth Responses and Biomass Allocation of Minmu 6 Under Cd and Pb Stress

Plant height, stem diameter, leaf dimensions, and root, stem, and leaf biomass were compared among treatments to characterize growth responses to Cd and Pb.

#### 2.1.1. Cd Stress Markedly Inhibited Shoot Growth and Biomass Accumulation

**Figure 1:**
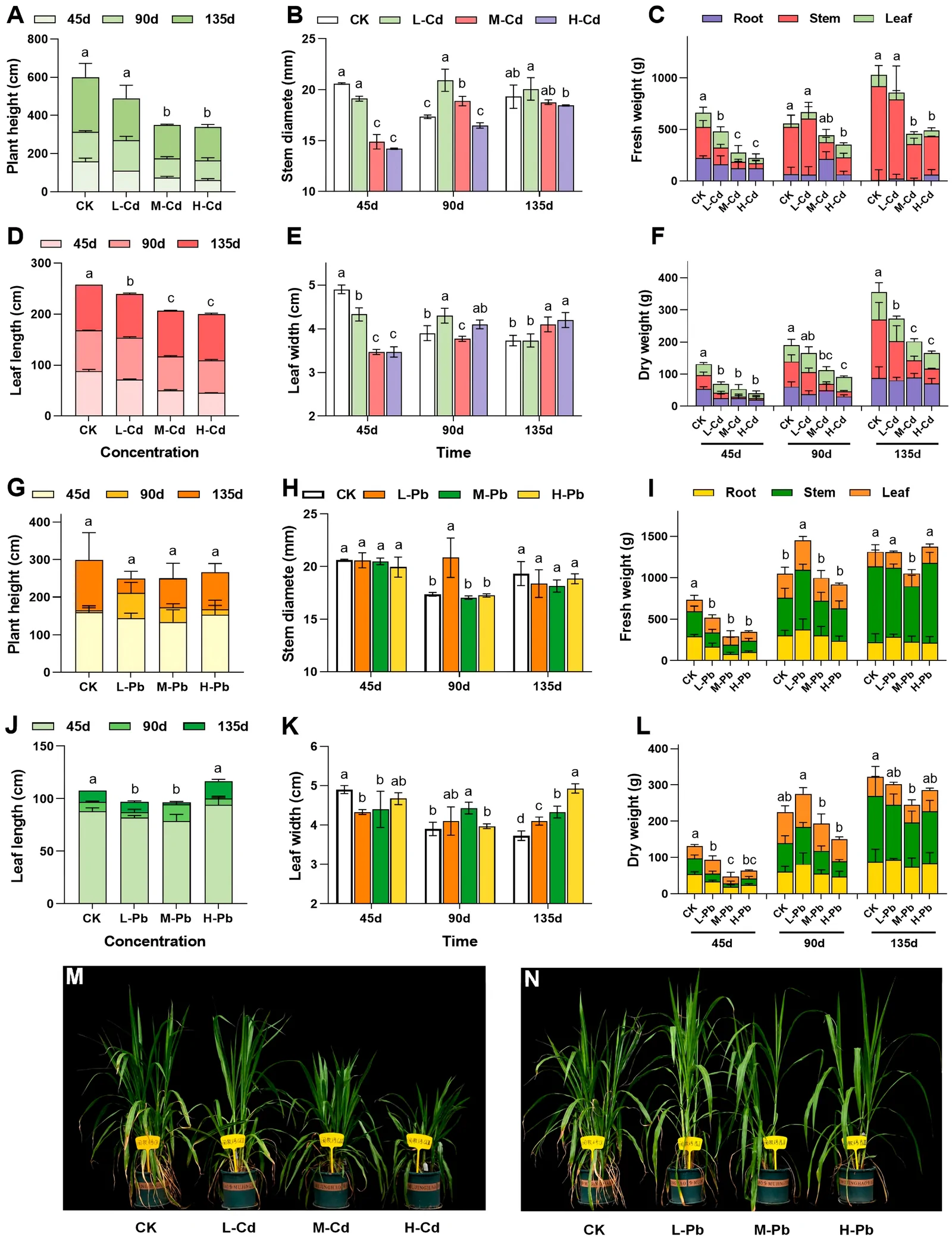
Growth and biomass allocation of Minmu 6 under Cd and Pb stress. (**A**–**F**) Net plant height increment, stem diameter, plant fresh weight, leaf length, leaf width, and dry weight under Cd stress, respectively. (**G**–**L**) The corresponding traits under Pb stress. (**M**,**N**) Representative growth phenotypes of Minmu 6 at 45 d under Cd and Pb treatments, respectively. Values are means ± standard error (SE; *n* = 3). Different lowercase letters indicate significant differences among treatments at the same sampling time, as determined by Tukey’s honestly significant difference (HSD) test (*p* < 0.05).

Plant height, stem diameter, and biomass varied with Cd concentration and sampling time (Figure 1A–F). At 45 d, plant height was lower in all Cd treatments than in the untreated control (CK). Heights under low (L-Cd, 1.50 mg kg^−1^), medium (M-Cd, 10.00 mg kg^−1^), and high (H-Cd, 25.00 mg kg^−1^) Cd treatments were 110.00, 73.00, and 60.67 cm, respectively, compared with 159.67 cm in CK. These values represented reductions of 31.10–62.00%. At 135 d, plant height remained lower in all Cd treatments than in CK (287.33 cm). Heights under M-Cd and H-Cd were similar, at 176.33 and 174.67 cm, respectively, indicating persistent growth inhibition at these concentrations.

Cd treatment also reduced stem diameter. At 45 d, stem diameter under H-Cd was 31.10% lower than in CK, and the difference was significant according to the Tukey groupings in Figure 1B (*p* = 0.0001). At 135 d, H-Cd and CK shared a Tukey letter, indicating that no significant difference was detected. The organ-level biomass means nevertheless showed substantial reductions at the early harvest: stem fresh weight and stem dry weight under H-Cd were 15.10% and 11.49% of CK, respectively, whereas root dry weight was 38.31% of CK. These percentages describe numerical differences and do not establish a statistically tested difference in sensitivity among organs.

Leaf dimensions also changed under Cd stress (Figure 1D,E). At 45 d, leaf length under H-Cd was 45.33 cm, only 51.33% of the CK value. By 135 d, leaf length in the Cd treatments had increased to 85–90 cm, narrowing the difference from CK. Leaf width showed a different pattern: at 135 d, it was 4.20 cm under H-Cd and 3.73 cm in CK. These results indicate stronger inhibition of leaf elongation at the early sampling stage, with smaller differences in leaf dimensions later in the experiment.

#### 2.1.2. Pb Caused Less Growth Inhibition than Cd at the Concentrations Tested

At the concentrations tested, Pb caused less growth inhibition than Cd (Figure 1G–L). At 45 d, plant height under H-Pb was 153.67 cm, close to the CK value of 159.67 cm. At 135 d, heights under L-Pb, M-Pb, and H-Pb were 249.00, 250.00, and 266.00 cm, respectively. Although all were lower than CK (287.33 cm), the reductions were smaller than those observed under Cd treatment.

Biomass responses to Pb varied across sampling stages. At 90 d, stem and root fresh weights under L-Pb were 716.67 and 378.67 g, respectively, exceeding the corresponding CK values of 453.67 and 302.33 g. At 135 d, stem fresh weight was 667.33 under M-Pb and 960.00 g under H-Pb, compared with 909.33 g in CK. Root dry weight under H-Pb was 83.67 g, close to the CK value of 87.33 g. Leaf length under H-Pb reached 104.00 cm at 135 d, compared with 90.00 cm in CK, while leaf width remained similar or increased slightly. Overall, Minmu 6 maintained substantial biomass under Pb treatment, particularly at the later sampling stage. Stem diameter did not differ significantly among Pb treatments at 45 or 135 d (Figure 1H), whereas L-Pb exceeded CK at 90 d (Tukey’s HSD, *p* = 0.0033).

### 2.2. Differential Physiological Responses of Minmu 6 Under Cd and Pb Stress

**Figure 2:**
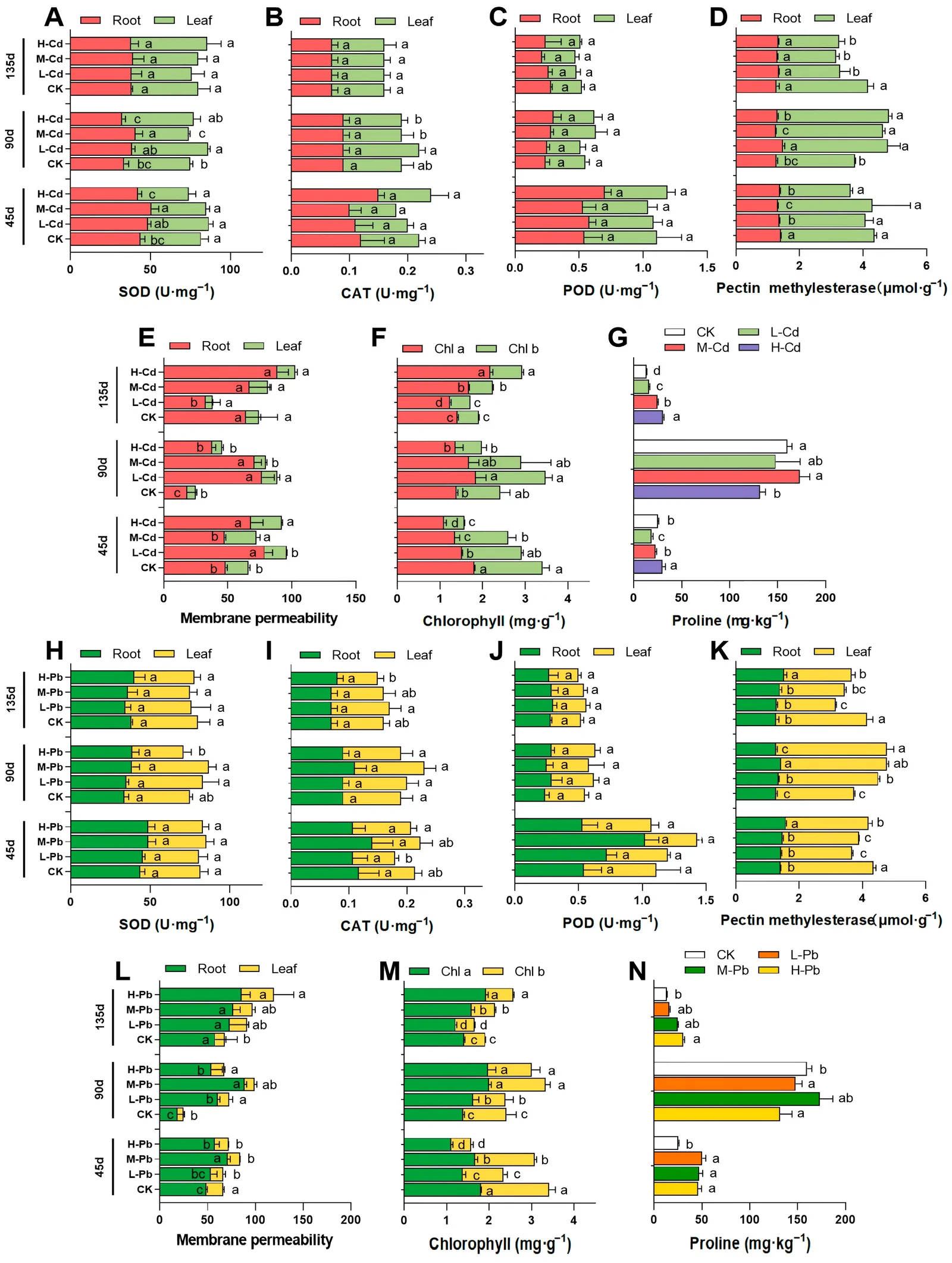
Physiological and biochemical responses of Minmu 6 under Cd and Pb stress (**A**–**G**) Changes in superoxide dismutase (SOD), catalase (CAT), and peroxidase (POD) activities, as well as pectin methylesterase content, membrane permeability, chlorophyll content, and proline content under Cd stress; (**H**–**N**) changes in SOD, CAT, and POD activities, as well as pectin methylesterase content, membrane permeability, chlorophyll content, and proline content under Pb stress. Data are presented as means ± SE (*n* = 3). Different lowercase letters indicate significant differences among treatments within the same sampling stage according to Tukey’s HSD test (*p* < 0.05).

Antioxidant enzyme activities, membrane permeability, cell wall pectin methylesterase content, photosynthetic pigments, and proline content were compared to characterize physiological responses to Cd and Pb (Figure 2).

#### 2.2.1. Antioxidant Enzyme Activities Varied with Metal Treatment, Organ, and Sampling Time

Under Cd treatment, leaf superoxide dismutase (SOD) activity showed numerical differences across treatments and sampling times, but no significant treatment differences were detected at 45 or 135 d (Figure 2A). At 45 d, the H-Cd mean was 31.64 U mg^−1^, compared with 37.80 U mg^−1^ in CK; at 135 d, the corresponding means were 47.51 and 41.83 U mg^−1^ in CK. Root SOD activity was highest under M-Cd at 45 d, reaching 50.74 U mg^−1^, 15.80% above CK, and the Tukey groupings indicated a significant difference (*p* = 0.0275). Thus, the numerical changes in leaf SOD should be distinguished from the statistically supported root response.

Under Pb stress, leaf SOD activity reached relatively high levels at 90 d. Values under L-Pb and M-Pb were 48.03 and 48.16 U mg^−1^, respectively, compared with 41.08 U mg^−1^ in CK. By 135 d, values in all Pb treatments were similar to or lower than CK. No clear increase relative to CK was observed under H-Pb at any sampling stage. Higher leaf SOD activity under Pb treatment was therefore mainly observed at 90 d.

Peroxidase (POD) activity differed between organs. At 45 d, mean root POD activity under H-Cd was 0.70 U mg^−1^, compared with 0.54 U mg^−1^ in CK, whereas leaf POD activity varied little among Cd treatments. Pb also increased root POD activity at 45 d; the value under M-Pb was 1.02 U mg^−1^, or 1.89 times the CK value. By 135 d, root POD activity in the Pb treatments was close to CK. These observations indicate that the early root POD response was greater under M-Pb than under.

#### 2.2.2. Membrane Permeability and Cell Wall Pectin Methylesterase Responses Differed Between Organs

Under Cd treatment, root membrane permeability differed among treatments at some sampling times (Figure 2E). At 45 d, the H-Cd value was 68.20%, compared with 48.45% in CK, with a significant difference according to the Tukey groupings (*p* = 0.0024). At 135 d, the means were 88.89% and 64.31%, respectively. At 45 d, leaf membrane permeability under M-Cd and H-Cd was 24.82% and 24.02%, respectively, and both exceeded CK (17.87%; *p* = 0.0003).

Under Pb treatment, root membrane permeability increased with both exposure duration and treatment concentration. Under H-Pb, it rose from 57.73% at 45 d to 85.46% at 135 d; the CK value at 135 d was 57.83%. Differences in leaf membrane permeability were also evident at 135 d, when H-Pb reached 34.01%, compared with 10.10% in CK. Thus, Pb-related changes in membrane permeability were more pronounced at the later sampling stage.

At 90 d, leaf cell wall pectin methylesterase content under H-Cd was 3.51 μmol g^−1^, compared with 2.46 μmol g^−1^ in CK (Figure 2D; Tukey’s HSD, *p* = 0.0001). Root pectin methylesterase means under Cd treatment ranged from 1.28 to 1.48 μmol g^−1^ across treatments and sampling times. Under Pb treatment, root pectin methylesterase content increased with treatment concentration at 135 d was 1.52 μmol g^−1^ under H-Pb and 1.28 μmol g^−1^ in CK, an 18.81% increase supported by the Tukey groupings (Figure 2K; *p* = 0.0070).

#### 2.2.3. Photosynthetic Pigments and Proline Responded Differently to Cd and Pb

Cd treatment markedly altered chlorophyll composition. At 135 d, chlorophyll a content under H-Cd was 2.18 mg g^−1^, compared with 1.41 mg g^−1^ in CK. At 45 d, chlorophyll b content under H-Cd was 0.47 mg g^−1^, below the CK value of 1.60 mg g^−1^, it increased slightly at later sampling stages. Under H-Pb, chlorophyll a and chlorophyll b contents at 135 d were 1.93 and 0.64 mg g^−1^, respectively, and both exceeded CK according to the Tukey groupings (Figure 2M; *p* = 0.0001). These comparisons concern individual pigments at specified sampling times and do not establish changes in photosynthetic performance.

Proline content varied across sampling times (Figure 2G,N). Under Cd treatment, the treatment means ranged from 131.57 to 173.10 mg kg^−1^ at 90 d and from 13.56 to 30.76 mg kg^−1^ at 135 d. At 90 d, H-Cd was lower than CK according to the Tukey groupings (*p* = 0.0371), whereas L-Cd and M-Cd did not differ significantly from CK. Under Pb treatment, proline contents at 45 d were 50.32, 47.21, and 46.17 mg kg^−1^ under L-Pb, M-Pb, and H-Pb, respectively, compared with 25.19 mg kg^−1^ in CK; all three treatments exceeded CK (*p* = 0.0001).

### 2.3. Metal Accumulation, Translocation, and Associated Soil Changes

#### 2.3.1. Cd Accumulated Mainly in Roots, with Partial Translocation to Shoots

**Figure 3:**
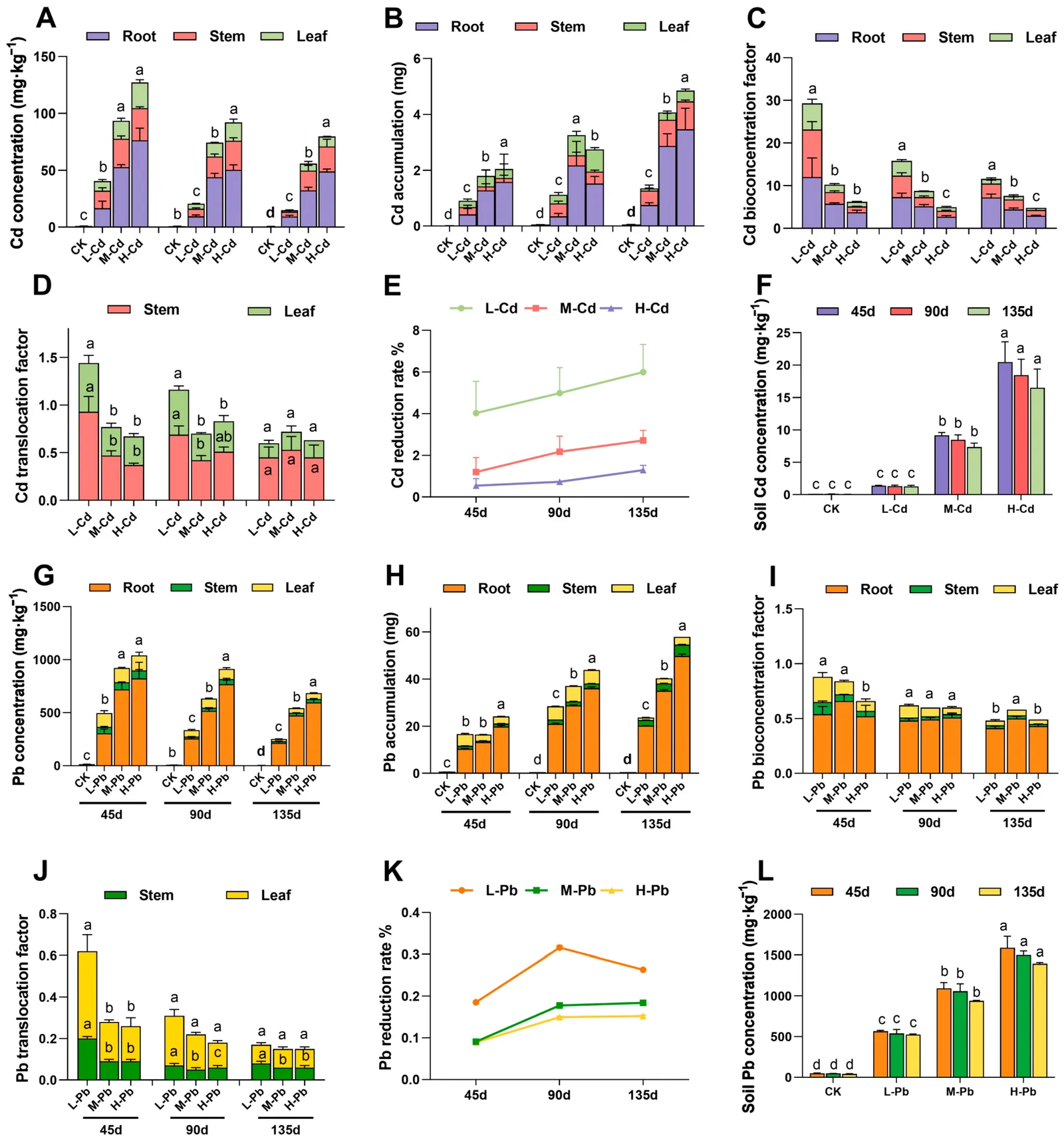
Cd and Pb accumulation, translocation, and associated soil changes in Minmu 6. (**A**–**F**) Cd concentration in roots, stems, and leaves; organ-level Cd accumulation; Cd bioconcentration factor (BCF); Cd translocation factor (TF); whole-plant Cd removal efficiency and soil Cd concentration, respectively. (**G**–**L**) The corresponding measurements for Pb. Values are means ± standard error (SE; *n* = 3). Where shown, different lowercase letters indicate significant differences among treatments at the same sampling time, as determined by Tukey’s honestly significant difference (HSD) test (*p* < 0.05).

Cd concentration followed the order root > stem > leaf (Figure 3A). At 45 d, root Cd concentration under H-Cd was 76.38 mg kg^−1^, or 2.71 and 3.38 times the concentrations in stems (28.17 mg kg^−1^) and leaves (22.59 mg kg^−1^), respectively. At 135 d, the corresponding concentrations were lower, at 48.87, 22.02, and 8.83 mg kg^−1^, with leaves showing the largest proportional decline.

Cd BCFs were highest under low Cd exposure (Figure 3C). At 45 d, root, stem, and leaf BCFs under L-Cd were 12.01, 11.13, and 6.15, respectively. BCFs decreased as the applied Cd concentration increased, reaching 3.73, 1.38, and 1.10, respectively, under H-Cd. At 135 d, root and leaf BCFs under H-Cd were 2.96 and 0.53. Root BCFs above 1, together with lower shoot BCFs and TFs below 1, indicate preferential accumulation in roots and restricted translocation. High root BCFs alone therefore do not demonstrate effective phytoextraction.

Cd TFs varied with concentration and sampling time (Figure 3D). At 45 d, the stem TF was 0.93 under L-Cd and 0.37 under H-Cd. At 135 d, stem TFs were 0.45, 0.53, and 0.45 under L-Cd, M-Cd, and H-Cd, respectively, while leaf TFs ranged from 0.18 to 0.19. These concentration ratios indicate lower Cd concentrations in aboveground tissues than in roots, particularly in leaves.

Organ-level accumulation and soil Cd concentration are shown in Figure 3B,F. Aboveground Cd accumulation represents the amount potentially removable by cutting, whereas root Cd accumulation represents belowground retention. At 135 d, whole-plant Cd removal efficiencies under H-Cd, M-Cd, and L-Cd were 1.29%, 2.71%, and 6.00%, respectively (Figure 3E). Thus, the Cd retained in the whole plant accounted for only a small proportion of the amount added to the soil, despite relatively high root Cd concentrations.

#### 2.3.2. Pb Accumulated Predominantly in Roots, with Limited Translocation to Shoots

Pb was more strongly concentrated in roots than Cd (Figure 3A,G). At 135 d under H-Pb, Pb concentrations were 595.20, 33.72, and 55.55 mg kg^−1^ in roots, stems, and leaves, respectively. Root concentrations were therefore 17.7 and 10.7 times those in stems and leaves, respectively. Across all Pb treatments and sampling stages, stem concentrations remained below 100 mg kg^−1^ and leaf concentrations below 150 mg kg^−1^. Root Pb concentrations increased with the applied Pb concentration.

Pb BCFs were generally lower than Cd BCFs (Figure 3C,I). The highest root Pb BCF was 0.54 under L-Pb at 45 d, whereas stem and leaf BCFs generally remained below 0.12. Pb TFs also indicated limited translocation to shoots (Figure 3J). Stem TFs did not exceed 0.20 and declined to 0.06–0.09 by 135 d; leaf TFs at 135 d were approximately 0.09. These low shoot-to-root concentration ratios are consistent with preferential Pb retention in roots.

Organ-level Pb accumulation and soil Pb concentration are shown in Figure 3H,L. Aboveground accumulation represents the potentially harvestable Pb fraction. At 135 d, whole-plant Pb removal efficiencies under H-Pb, M-Pb, and L-Pb were only 0.21%, 0.22%, and 0.26%, respectively (Figure 3K). These values indicate limited recovery of added Pb in plant tissues. Together with low shoot-to-root concentration ratios, they suggest that the potential value of Minmu 6 under Pb stress is more closely related to root retention than to harvestable phytoextraction.

#### 2.3.3. Soil Cd and Pb Concentrations Across Harvest Stages

The initial background concentrations of Cd and Pb in the composite soil before metal addition were 0.16 and 50.63 mg kg^−1^, respectively. Total soil Cd concentrations remained clearly separated among the Cd treatments throughout the experiment (Figure 3F). At 45 d, soil Cd concentrations were 0.14, 1.38, 9.16, and 20.46 mg kg^−1^ in CK, L-Cd, M-Cd, and H-Cd, respectively; by 135 d, the corresponding values were 0.05, 1.29, 7.35, and 16.51 mg kg^−1^. Thus, total soil Cd concentrations showed a general decline with sampling time, particularly in the M-Cd and H-Cd treatments.

A similar temporal pattern was observed for Pb (Figure 3L). Soil Pb concentrations at 45 d were 49.85, 567.75, 1093.16, and 1588.27 mg kg^−1^ in CK, L-Pb, M-Pb, and H-Pb, respectively, and decreased to 40.98, 524.96, 940.19, and 1394.59 mg kg^−1^ at 135 d. Despite these temporal decreases, the concentration gradients among treatments remained distinct throughout the experiment. Because different pots were destructively sampled at each harvest, these temporal differences represent treatment-level patterns across independent harvest groups rather than repeated measurements of the same soil.

### 2.4. Mantel Associations Among Tissue Metal Concentrations and Plant Traits

Mantel tests were used to explore associations among the applied metal concentration, tissue metal concentrations, exposure duration, and plant traits. These tests assess correlations between distance matrices, which summarize pairwise differences among samples. They were used to identify patterns of covariation, not to infer causal relationships.

#### 2.4.1. Tissue Cd Concentrations Were Associated with Growth and Physiological Traits

**Figure 4:**
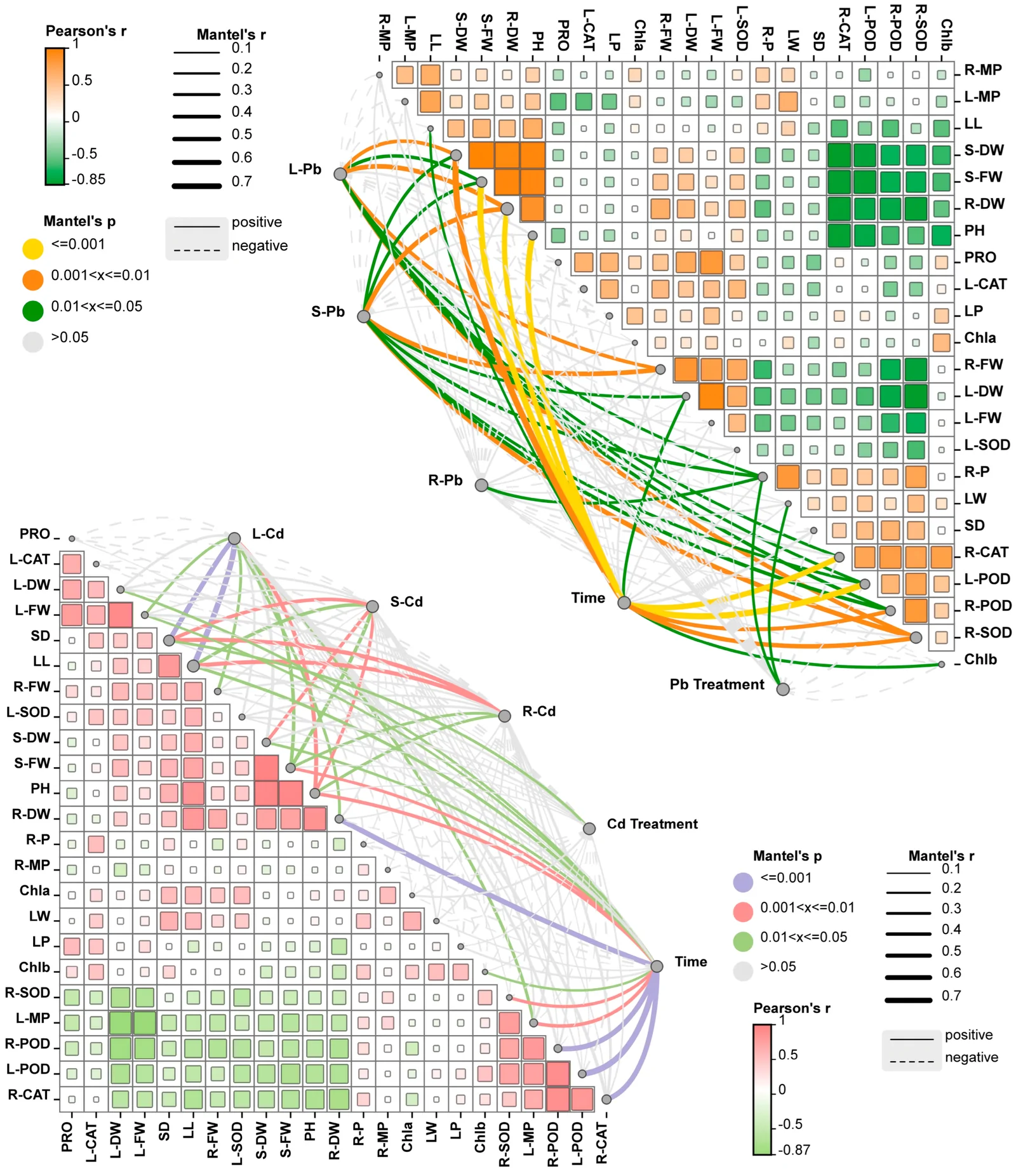
Mantel associations among tissue metal concentrations, exposure duration, and plant traits. Lines show Mantel associations of the applied metal concentration, organ-specific metal concentrations, and exposure duration with growth or physiological traits. Line color indicates statistical significance, and line width indicates the magnitude of the Mantel correlation coefficient. Heatmap colors represent Pearson correlation coefficients.

Several significant Mantel associations were detected between tissue Cd concentrations and plant traits (Figure 4). Leaf Cd concentration was associated with leaf length (*r* = 0.744, *p* = 0.001), stem diameter (*r* = 0.628, *p* = 0.001), plant height (*r* = 0.501, *p* = 0.007), and the fresh and dry weights of roots, stems, and leaves (*p* < 0.05). It was also associated with leaf membrane permeability (*r* = 0.334, *p* = 0.036). Stem Cd concentration was associated with stem diameter, stem fresh and dry weights, and plant height (*r* = 0.368–0.425, *p* < 0.05). Root Cd concentration also showed significant associations with leaf length and stem diameter.

Exposure duration was associated with root CAT activity (*r* = 0.680, *p* = 0.001), POD activity (*r* = 0.661, *p* = 0.001), and SOD activity (*r* = 0.361, *p* = 0.009). Associations were also detected for leaf POD activity (*r* = 0.798, *p* = 0.001) and membrane permeability (*r* = 0.411, *p* = 0.007). By contrast, the applied Cd concentration was associated only with stem diameter (*r* = 0.289, *p* = 0.049), with no significant associations detected for most physiological traits. More nominally significant associations were detected for tissue Cd concentrations and exposure duration than for the applied concentration in this dataset. These findings do not establish tissue Cd concentration as an independent driver of those responses.

#### 2.4.2. Exposure Duration and Tissue Pb Concentrations Were Associated with Biomass Traits

Under Pb stress, exposure duration was associated with plant height (*r* = 0.750, *p* = 0.001), stem fresh weight (*r* = 0.794, *p* = 0.001), stem dry weight (*r* = 0.790, *p* = 0.002), and root dry weight (*r* = 0.660, *p* = 0.001). It was also associated with root CAT (*r* = 0.687, *p* = 0.001), SOD (*r* = 0.532, *p* = 0.004), and POD activities (*r* = 0.456, *p* = 0.008), leaf POD activity (*r* = 0.789, *p* = 0.001), and chlorophyll b content (*r* = 0.303, *p* = 0.024) (Figure 4).

Associations between tissue Pb concentrations and biomass differed among organs. Leaf Pb concentration was associated with stem fresh weight (*r* = 0.371, *p* = 0.021), stem dry weight (*r* = 0.405, *p* = 0.010), root fresh weight (*r* = 0.403, *p* = 0.007), and root dry weight (*r* = 0.566, *p* = 0.005). Root Pb concentration was associated with stem fresh weight (*r* = 0.335, *p* = 0.026) and stem dry weight (*r* = 0.339, *p* = 0.022), but not with root SOD (*r* = 0.155, *p* = 0.104) or POD activity (*r* = −0.017, *p* = 0.521). The applied Pb concentration showed only weak associations with leaf width (*r* = 0.247, *p* = 0.043) and root pectin methylesterase content (*r* = 0.251, *p* = 0.046). In this dataset, more nominally significant associations involved exposure duration and tissue Pb concentrations than the applied Pb concentration; this pattern is descriptive and does not imply causal priority.

## 3. Discussion

The results are consistent with the working hypothesis of contrasting Cd and Pb partitioning under the selected exposure regimes: Cd showed greater transfer to shoots, whereas Pb remained more strongly associated with roots. However, this pattern does not by itself establish different remediation functions. The practical distinction depends on the amount of metal recoverable through harvesting and, for stabilization, evidence that soil metal mobility or exposure is reduced.

### 3.1. Biomass and Shoot Cd Accumulation Must Be Evaluated Together

Minmu 6 accumulated Cd in stems and leaves, but the associated growth reductions may constrain the amount removable through harvesting. High-biomass grasses can contribute to phytoextraction when shoot biomass compensates for moderate tissue metal concentrations [4]. In this experiment, however, neither a high root BCF nor the persistence of some shoot biomass demonstrates efficient extraction. The relevant endpoint is harvestable Cd recovery, calculated from shoot Cd concentration and dry biomass.

The field experiment of Zhou et al. [6,16,17] provides a useful comparison because it evaluated king grass in several soil types with Cd that had aged for 6 years and compared harvesting at elongation and maturity. The present experiment instead used one soil and a 7 d equilibration period. Differences in soil aging, substrate properties, and harvest stage limit direct extrapolation of field extraction performance to Minmu 6. Within the present dataset, stem and leaf Cd accumulation supports further assessment of harvestable removal, but whole-plant metal removal efficiencies of 1.29–6.00% at 135 d include root-associated Cd. Shoot-based recovery must therefore be lower than the corresponding whole-plant values. BCF and TF are therefore useful descriptors of accumulation and partitioning, but they should not be interpreted independently of organ identity and biomass. Recent comparisons among crop species likewise showed substantial organ- and species-specific variation in Cd BCF and TF, reinforcing the need to interpret these indices in their biological context [18].

The whole-plant Cd removal efficiencies observed here (1.29–6.00% at 135 d) are low. Moreover, because these values include root-associated Cd, the fraction actually removable by conventional shoot harvesting is necessarily lower. Thus, the present results do not indicate rapid stand-alone phytoextraction. This limitation is consistent with earlier work on king grass: despite high annual biomass production and measurable Cd extraction, Zhang et al. [10] estimated that remediation of soils containing 8–100 mg kg^−1^ Cd could require approximately 23–290 years, illustrating that high biomass alone does not guarantee rapid soil decontamination.

The field study of Zhou et al. [17] provides a more specific comparison. The comparable findings are the root-biased Cd distribution, BCF values > 1, TF values < 1, and the observation that substantial shoot biomass can still generate measurable harvestable Cd removal despite preferential root accumulation. However, absolute extraction efficiencies are not directly comparable: Zhou et al. used three field soil types containing Cd that had aged for six years, Cd treatments of 0.25–16 mg kg^−1^, and two harvest stages, whereas the present study used one red soil, a 7 d equilibration period, and higher experimental Cd additions. Zhou et al. also reported that shoot Cd extraction was related to DTPA-extractable soil Cd and harvest stage, variables not measured here [17].

A similar distinction emerges from the pot-and-field study of hybrid *Pennisetum* by Wu et al. [16] Under pot conditions, root BCF exceeded 1, shoot BCF remained <1, and TF was <0.5, whereas in lightly Cd-contaminated field soil the very high aboveground biomass allowed shoots to account for most of the plant-extracted Cd. Together, these studies indicate that BCF and TF should be interpreted alongside harvestable biomass and field soil availability rather than used alone to infer practical extraction efficiency.

Physiological responses should also be interpreted in relation to their statistical support and functional specificity. The increase in chlorophyll a at 135 d and reduction in chlorophyll b at 45 d occurred at different sampling times and do not establish an altered chlorophyll a/b ratio or improved photosynthesis. Likewise, numerical changes in leaf SOD and root POD were not consistently significant. Pigment and enzyme measurements therefore describe responses associated with exposure, rather than demonstrating a coordinated detoxification mechanism. Gas-exchange measurements and direct assays of oxidative injury would be needed to establish their functional consequences.

### 3.2. Root-Associated Pb Does Not by Itself Demonstrate Phytostabilization

Pb was retained more strongly in roots and translocated less readily to shoots than Cd. Under Pb treatment, root Pb concentrations were markedly higher than those in stems and leaves. Stem TFs did not exceed 0.20, and leaf TFs were approximately 0.09 at 135 d. These low concentration ratios are consistent with limited Pb translocation, but they do not quantify the fraction of absorbed Pb transferred to shoots. The observed distribution agrees with reports of low Pb mobility and retention at root surfaces, in cell walls, and in the rhizosphere [11,12]. However, this root-biased distribution does not itself demonstrate phytostabilization. Rather, it identifies a root-retention phenotype that may be relevant to phytostabilization and requires independent confirmation through measurements of soluble or bioavailable Pb, leaching, or other indices of soil Pb mobility.

Changes in root POD activity and pectin methylesterase content, together with preferential root Pb accumulation, suggest that cell wall processes may contribute to Pb retention. However, cell wall-bound Pb and pectin methylesterase methylesterification, a property that affects pectin methylesterase charge and metal binding, were not measured. The proposed mechanism therefore requires verification through subcellular fractionation and direct analysis of cell wall-associated Pb.

Phytostabilization requires evidence of reduced contaminant mobility or exposure, rather than root accumulation alone. The whole-plant Pb removal efficiencies of only 0.21–0.26% at 135 d are particularly low and show that plant uptake accounted for only a very small fraction of the nominally added Pb. The experiment did not establish whether planting reduced soluble or bioavailable Pb, leaching losses, or transfer to other environmental compartments. Root retention therefore identifies a trait of interest for further study, but neither this trait nor a decline in total soil Pb concentration demonstrates successful stabilization.

### 3.3. Harvesting and Metal Mass Balance Determine Practical Relevance

Tissue metal concentration, BCF, or TF measured at a single time point cannot fully describe the remediation potential of a high-biomass grass. Assessment must also consider growth stage, biomass production, metal partitioning among organs, and the harvesting strategy [19]. This interpretation is supported by recent field work with perennial biomass crops: Suo et al. [20] found that roots were the principal metal-accumulating organs of miscanthus and switchgrass, while Cd and Pb partitioning changed across growth stages. Thus, the timing of sampling or harvest can materially alter the apparent remediation value inferred from organ concentrations alone.

*Sedum alfredii* Hance provides a useful comparison because it can accumulate Cd and Pb [21]. The comparison is informative not because Minmu 6 behaves as a hyperaccumulator, but because it illustrates how metal identity changes the meaning of organ-level accumulation. A meta-analysis of *S. alfredii* and *S. plumbizincicola* showed that Cd and Zn accumulation was generally greater in shoots than in roots, whereas Pb accumulated preferentially in roots; accumulation was also influenced by soil pH and organic matter [22]. Taylor & Francis Online Direct synchrotron analyses of *S. alfredii* further demonstrated spatially restricted Pb localization [21]. The relevant lesson for Minmu 6 is therefore that root-dominated Pb accumulation and greater Cd transfer to shoots can reflect metal-specific partitioning, but neither pattern alone establishes remediation performance. For Cd, practical extraction still requires sufficient metal accumulation in harvestable shoots; for Pb, root retention must be accompanied by demonstrable reduction in soil metal mobility before phytostabilization can be claimed.

Studies of hybrid *Pennisetum* highlight the need to evaluate biomass and harvesting strategy alongside tissue metal concentrations [16]. Chen et al. [6] likewise showed that root retention, movement of Cd among soil layers, and biomass production affect assessments of remediation performance. Recent crop comparisons also demonstrate that BCF and TF can differ substantially among species and organs under the same contaminated environment [18], while a global synthesis of 547 observations showed that metal identity, soil properties, plant traits, and biomass jointly influence bioaccumulation and translocation [23]. For Minmu 6, Cd phytoextraction potential should therefore be evaluated from the amount of Cd recoverable in harvested shoots, together with the capacity to maintain biomass under contamination.

Broader assessments also show that remediation performance depends on metal identity, soil properties, plant traits, metal accumulation, translocation, and harvestable biomass [23,24]. For Cd, evaluation should combine shoot Cd accumulation, biomass maintenance, and harvest timing. For Pb, root retention and restricted shoot translocation are relevant, but reduced soil mobility must be demonstrated independently. Changes in soil metal concentration should be interpreted alongside a metal mass balance. The Mantel results support consideration of exposure duration, although they describe covariation rather than causal effects.

Taken together, the findings identify differences in Cd and Pb partitioning that can guide further testing of Minmu 6. Demonstration of practical remediation performance will require separate evidence for harvestable metal removal and for reduced soil metal mobility or exposure.

### 3.4. Limitations and Future Directions

Several limitations affect the interpretation of these results. The 7 d equilibration period does not reproduce long-term aging in field soils and may therefore limit the representativeness of metal availability. Three biological replicates per treatment may also provide limited power to detect small effects. The proposed role of root cell walls in Pb retention is based on indirect evidence and requires direct analysis of subcellular metal distribution and cell wall-bound Pb [22]. In addition, cation exchange capacity (CEC), electrical conductivity (EC), soil pH dynamics during cultivation, and bioavailable metal fractions were not monitored. The absence of these measurements limits interpretation of metal availability and soil chemical changes; a complete metal mass balance is also needed to explain changes in total soil concentrations. Cd and Pb were tested separately, so the results cannot predict their interactions in co-contaminated soils. Finally, the Mantel tests are exploratory. Future studies should combine time-resolved sampling with experiments designed to test specific causal mechanisms.

The low whole-plant removal efficiencies observed here—1.29–6.00% for Cd and 0.21–0.26% for Pb—also impose an important practical limitation. Under the present conditions, Minmu 6 should therefore not be considered a stand-alone technology for rapid removal of soil metals. A more realistic role is as one component of an integrated remediation strategy. For Cd, this could include repeated biomass harvesting combined with carefully controlled measures that improve plant growth or Cd transfer to harvestable tissues; for Pb, vegetation-based root retention would need to be coupled with approaches that demonstrably reduce soil Pb mobility and with long-term monitoring [25].

Evidence from hybrid *Pennisetum* supports this integrated approach. He et al. [26] showed that digestate application increased *Pennisetum hybridum* biomass, rhizosphere available Cd, and root-to-shoot Cd transfer, and shortened the estimated remediation period from 15–16 years to approximately 10 years under an assumed three harvests per year. Such enhancement strategies must nevertheless be evaluated together with metal mobility and leaching risk, because increasing Cd availability may improve extraction while simultaneously increasing environmental exposure. Recent evidence from Napier grass further supports the potential value of assisted phytomanagement. Maung et al. [27] reported that salicylic acid treatment enhanced biomass production and Cd-Pb accumulation in *Pennisetum purpureum* cv. Mahasarakham under combined Cd–Pb contamination, with a higher bioaccumulation potential for Cd than for Pb. Their results indicate that plant-growth-regulator-assisted management may improve both plant performance and metal retention or uptake in Napier grass. However, because that study used a different cultivar, combined Cd-Pb contamination, and salicylic acid treatment, its reported phytostabilization performance should not be directly extrapolated to untreated Minmu 6.

Within these limits, Minmu 6 is better regarded as a candidate component of integrated Cd/Pb management than as an independently sufficient remediation technology. Its high biomass and contrasting organ-level partitioning provide useful traits for further optimization, but field-scale performance should be established in aged contaminated soils using direct measurements of harvestable removal, bioavailable metal pools, and metal mass balance.

## 4. Materials and Methods

### 4.1. Plant Material and Soil Conditions

Minmu 6 was developed by the Fujian Academy of Agricultural Sciences. Soil was collected from the cultivated layer at multiple points within the same field in Minhou, Fujian Province, China (25°57′ N, 119°18′ E), and combined into a composite batch. The soil was air-dried in the shade, roots and stones were removed, and the soil was thoroughly homogenized. Before metal addition and fertilization, a composite subsample was collected to determine the initial soil properties. Background Cd and Pb concentrations were 0.16 and 50.63 mg kg^−1^, respectively, and soil pH was 6.1. Organic matter, total nitrogen, total phosphorus, and total potassium contents were 21.2, 1.7, 10.7, and 21.8 g kg^−1^, respectively.

### 4.2. Experimental Design

The pot experiment followed a completely randomized design, with destructive sampling at 45, 90, and 135 d. Each pot contained 15 kg of soil on a dry-weight basis and received urea [CO(NH_2_)_2_] (3.26 g pot^−1^), single superphosphate (5.00 g pot^−1^), and potassium sulfate (1.50 g pot^−1^) as basal fertilizers. These applications supplied approximately 1.50 g N, 0.60 g P_2_O_5_, and 0.75 g K_2_O per pot, corresponding to an N:P_2_O_5_:K_2_O ratio of approximately 10:4:5. No additional fertilizer was applied during the entire cultivation period. We used analytical-grade CdCl_2_ as the exogenous Cd source and guaranteed-reagent-grade PbCl_2_ as the exogenous Pb source. The brand of these the reagents mentioned is Solarbio (China, Beijing). Before planting, the metal solutions and basal fertilizers were thoroughly mixed into the soil, which was then equilibrated under natural conditions for 7 d.

Cd and Pb treatments were established separately at each sampling stage. Cd addition rates were 0, 1.5 mg kg^−1^ (L-Cd), 10 mg kg^−1^ (M-Cd), and 25 mg kg^−1^ (H-Cd), and Pb addition rates were 0, 600 mg kg^−1^ (L-Pb), 1200 mg kg^−1^ (M-Pb), and 1800 mg kg^−1^ (H-Pb). Pots had an upper diameter of 26.8 cm, a base diameter of 22.0 cm, and a height of 28.9 cm. Each contained 15 kg of soil on a dry-weight basis and one plant. Each treatment at each sampling stage comprised three biological replicates, giving 72 pots in total. Each pot received 1.5 L of tap water daily, measured using a graduated cylinder, and all treatments followed the same irrigation schedule. Plants were destructively harvested at 45, 90, and 135 d to measure growth, physiological traits, and metal accumulation.

### 4.3. Measurements

#### 4.3.1. Agronomic Traits and Biomass

Tiller number, plant height, leaf length, leaf width, and stem diameter were measured before each harvest. Plant height was measured from the stem base to the tip of the uppermost leaf using a measuring tape. Leaf length and width were measured on the third fully expanded leaf from the top, and stem diameter was measured at the middle of the second aboveground internode using a vernier caliper. Harvested plants were separated into roots, stems, and leaves. Roots were thoroughly rinsed with tap water and blotted dry before fresh weight was recorded. Samples were heated at 105 °C for 30 min and then dried at 65 °C to constant weight. Root, stem, and leaf dry weights were recorded, and water content was calculated.

#### 4.3.2. Physiological and Biochemical Parameters

Membrane permeability was assessed using a DDS-11A conductivity meter [28]. Chlorophyll was extracted with an ethanol–acetone mixture (1:2, *v*/*v*) [29], and chlorophyll a and b contents were calculated as follows:

Chl a = (13.95 × A_665_ − 6.88 × A_649_) × V/(1000 × W)

Chl b = (24.96 × A_649_ − 7.32 × A_665_) × V/(1000 × W)

Here A_665_ and A_649_ are the absorbances at 665 nm and 649 nm, respectively; V is the final volume of the extract (mL); and W is the fresh weight of the leaf sample (g). Chlorophyll content is expressed in mg g^−1^ fresh mass.

Cell wall fractions were extracted following the method described by Zhong and Läuchli [30]. Total pectin methylesterase (TP) in root cell walls was measured using a commercial total pectin assay kit (Solarbio, Beijing, China), with a calibration curve prepared from a series of galacturonic acid standards. SOD activity was determined spectrophotometrically. One unit was defined as the amount of enzyme required to inhibit nitroblue tetrazolium photoreduction by 50%. POD [31] and CAT [32] activities were also measured spectrophotometrically; one unit corresponded to an absorbance change of 0.01 min^−1^ at 470 and 240 nm, respectively.

#### 4.3.3. Metal Accumulation and Translocation

Plant tissue Cd and Pb concentrations were measured by flame atomic absorption spectrophotometry after HNO_3_ digestion [33]. Procedural blanks were included in each digestion batch for background correction, and each sample was analyzed in triplicate. Instrument calibration was checked with standard solutions before each analytical run.

At each harvest, soil was destructively sampled from each pot. The entire contents of each pot were emptied onto a disposable plastic sheet, and the plant was separated from the soil. After removal of remaining roots, stones, and other debris, the soil was thoroughly mixed. Five subsamples of approximately 100 g each were collected from points along the diagonals of the mixed soil and pooled to obtain one composite sample of approximately 500 g per pot. Clean sampling tools were used and gloves were changed before sampling each pot to minimize cross-contamination. Each pot provided one independent soil sample; the five subsamples were not treated as biological replicates.

Total soil Cd and Pb concentrations were measured by flame atomic absorption spectrophotometry after HNO_3_-HClO_4_ digestion. Quality control was performed using matrix spike recovery tests. The recoveries of Cd and Pb in soil samples were 91.56–93.21% and 92.06–94.77%, respectively, while those in plant samples were 93.51–95.22% for Cd and 94.10–96.68% for Pb, respectively.

The bioconcentration factor (BCF), translocation factor (TF), and whole-plant metal removal efficiency were calculated as follows:

BCF = metal concentration in the specified organ (mg kg^−1^)/soil metal concentration (mg kg^−1^)

TF = metal concentration in the specified aboveground organ (mg kg^−1^)/root metal concentration (mg kg^−1^)

Whole-plant metal removal efficiency (%) = [(root metal accumulation + stem metal accumulation + leaf metal accumulation)/total metal added to the soil] × 100%

Metal accumulation in each organ (mg) was calculated as tissue metal concentration (mg kg^−1^) multiplied by organ dry mass (kg). The amount of metal added to each pot (mg) was calculated as the nominal addition rate (mg kg^−1^) multiplied by the soil dry mass (15 kg). Whole-plant metal removal efficiency therefore expresses total plant-associated metal as a percentage of the added metal amount. It includes root-associated metal and does not quantify removal by shoot harvesting alone. The index is not defined for the zero-addition controls.

### 4.4. Data Analysis

Data were organized in Excel 2019 and analyzed in SPSS 26.0. Treatment differences were evaluated using one-way analysis of variance (ANOVA), followed by Tukey’s honestly significant difference (HSD) test when the ANOVA was significant. Statistical significance was set at *p* < 0.05, and results are reported as means ± standard error (SE). Pearson correlations were used to examine associations between metal stress and agronomic traits. Figures were prepared in GraphPad Prism 9.5. Mantel tests were used to evaluate distance–matrix associations of the applied metal concentration, organ-specific metal concentrations, and exposure duration with growth and physiological traits. Mantel analyses and associated visualizations were performed in R (V. 4.3.0) using the ggcor package.

## 5. Conclusions

Under the pot conditions tested, Minmu 6 showed contrasting growth responses and organ-level partitioning of Cd and Pb. Cd occurred in both roots and harvestable shoots, whereas Pb was concentrated predominantly in roots and had low shoot-to-root concentration ratios. These observations identify different targets for further investigation, rather than demonstrating metal-specific remediation functions. Whole-plant metal removal efficiencies were low and included root-associated metal; shoot harvesting would remove a smaller fraction. Root Pb retention alone does not establish phytostabilization, because reduced soil Pb mobility and environmental exposure were not demonstrated. Further assessment should quantify harvestable metal removal, soil metal availability, and metal mass balance in aged contaminated soils under field conditions. The present findings are limited to separate Cd and Pb treatments, a 7 d soil equilibration period, and exploratory associations among plant traits.

## Data Availability

The raw data supporting the conclusions of this article will be made available by the authors on request. Further inquiries can be directed to the corresponding authors.

## Abbreviations

The following abbreviations are used in this manuscript:

L-Cd	Low Cd treatment
M-Cd	Medium Cd treatment
H-Cd	High Cd treatment
L-Pb	Low Pb treatment
M-Pb	Medium Pb treatment
H-Pb	High Pb treatment
